# Redetermination of Nd_2_Ti_2_O_7_: a non-centrosymmetric structure with perovskite-type slabs

**DOI:** 10.1107/S1600536813005497

**Published:** 2013-03-06

**Authors:** Nobuo Ishizawa, Keisuke Ninomiya, Terutoshi Sakakura, Jun Wang

**Affiliations:** a Advanced Ceramics Research Center, Nagoya Institute of Technology, Asahigaoka 10-6-29, Tajimi 507-0071, Japan; b Institute of Multidisciplinary Research for Advanced Materials, Tohoku University, Katahira 2-1-1, Aoba-ku, Sendai 980-8577, Japan; c School of Materials Science & Engineering, Inner Mongolia University of Technology, Hohhot 010051, People’s Republic of China

## Abstract

Single crystals of dineodymium(III) dititanium(IV) hepta­oxide, Nd_2_Ti_2_O_7_, were synthesized by the flux method and found to belong to the family of compounds with perovskite-type structural motifs. The asymmetric unit contains four Nd, four Ti and 14 O-atom sites. The perovskite-type slabs are stacked parallel to (010) with a thickness corresponding to four corner-sharing TiO_6_ octa­hedra. The Nd and Ti ions are displaced from the geometrical centres of respective coordin­ation polyhedra so that the net polarization occurs along the *c* axis. The investigated crystals were all twinned and have a halved monoclinic unit cell in comparison with the first structure determination of this compound [Scheunemann & Müller-Buschbaum (1975 ▶). *J. Inorg. Nucl. Chem.* **37**, 2261–2263].

## Related literature

For previous determinations of Nd_2_Ti_2_O_7_, see: Scheunemann & Müller-Buschbaum (1975 ▶); Harvey *et al.* (2005 ▶). For related compounds, see: Gasperin (1975 ▶); Ishizawa *et al.* (1980 ▶); Schmalle *et al.* (1993 ▶). For the extinction method, see: Becker & Coppens (1974 ▶).

## Experimental

### 

#### Crystal data

Nd_2_Ti_2_O_7_
*M* *_r_* = 496.2
Monoclinic, 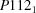

*a* = 7.6747 (1) Å
*b* = 13.0025 (2) Å
*c* = 5.4640 (1) Å
γ = 98.5165 (5)°
*V* = 539.24 (2) Å^3^
*Z* = 4
Mo *K*α radiation
μ = 21.77 mm^−1^
*T* = 293 K
0.11 × 0.08 × 0.07 mm

#### Data collection

Bruker APEXII CCD diffractometer
Absorption correction: multi-scan (*TWINABS*; Bruker, 2008 ▶) *T*_min_ = 0.134, *T*_max_ = 0.206
25187 measured reflections
6608 independent reflections
6200 reflections with *I* > 3σ(*I*)
*R*_int_ = 0.049

#### Refinement

*R*[*F* ^2^ > 2σ(*F* ^2^)] = 0.024
*wR*(*F* ^2^) = 0.027
*S* = 1.37
6608 reflections
133 parameters
Δρ_max_ = 2.44 e Å^−3^
Δρ_min_ = −1.57 e Å^−3^
Absolute structure: Flack (1983 ▶), 3019 Friedel pairs
Flack parameter: 0.220 (12)

### 

Data collection: *APEX2* (Bruker, 2008 ▶); cell refinement: *SAINT* (Bruker, 2008 ▶); data reduction: *SAINT*; program(s) used to solve structure: *SUPERFLIP* (Palatinus & Chapuis, 2007 ▶); program(s) used to refine structure: *JANA2006* (Petříček *et al.*, 2006 ▶); molecular graphics: *ATOMS* (Dowty, 2006 ▶) and *DIAMOND* (Brandenburg & Putz, 2005 ▶); software used to prepare material for publication: *PLATON* (Spek, 2009 ▶) and *publCIF* (Westrip, 2010 ▶).

## Supplementary Material

Crystal structure: contains datablock(s) global, I. DOI: 10.1107/S1600536813005497/wm2720sup1.cif

Structure factors: contains datablock(s) I. DOI: 10.1107/S1600536813005497/wm2720Isup2.hkl

Additional supplementary materials:  crystallographic information; 3D view; checkCIF report

## Figures and Tables

**Fig. 1.**
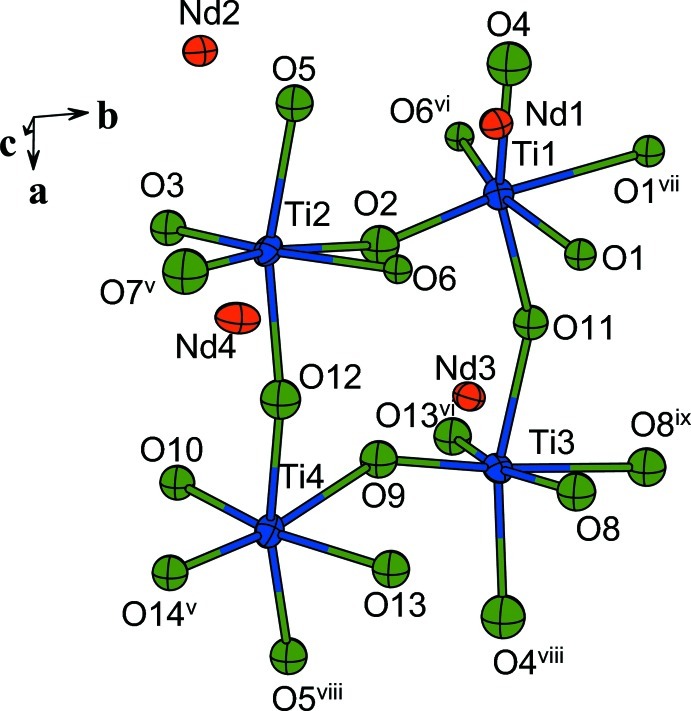
View of a part of the Nd2Ti2O7 structure with displacement ellipsoids drawn at the 95% probability level. Symmetry codes are given in the geometric parameters Table.

**Fig. 2.**
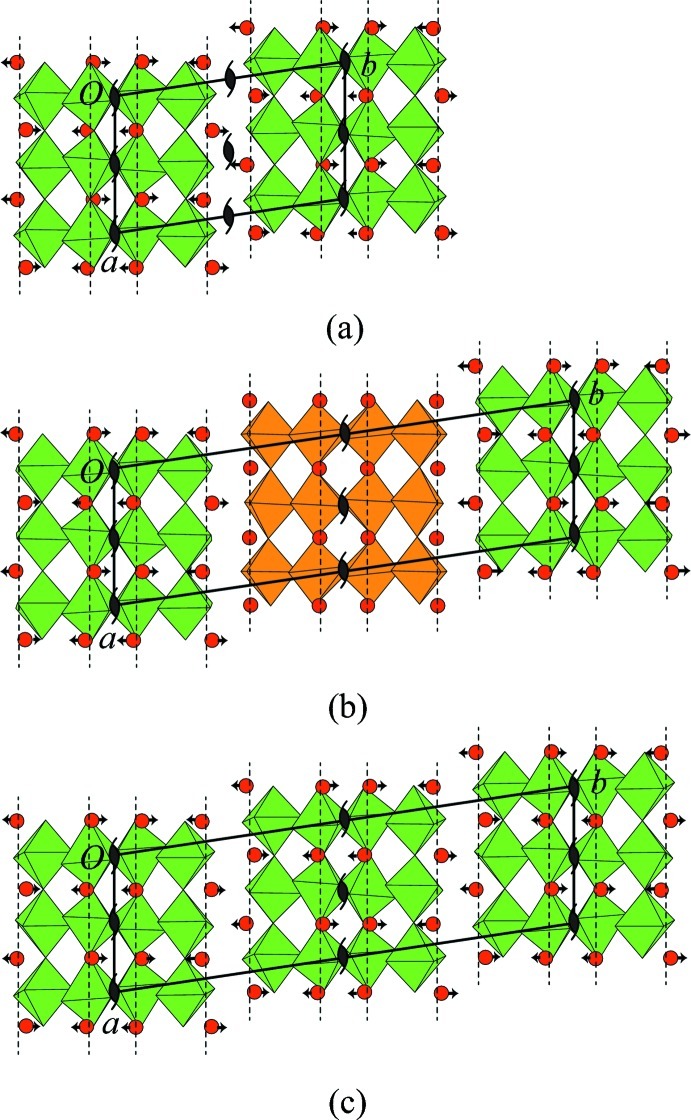
Comparison of the Nd2Ti2O7 structures, (a) present study, (b) Scheunemann & Müller-Buschbaum (1975), (c) Harvey et al. (2005). Unit cells and atomic parameters are shifted for comparison. Two kinds of perovskite-type slabs are illustrated using green- and orange-coloured TiO6 octahedra, respectively. The small black arrows indicate the displacements of Nd atoms (red spheres) along b* with respect to the dashed lines drawn parallel to a.

**Fig. 3.**
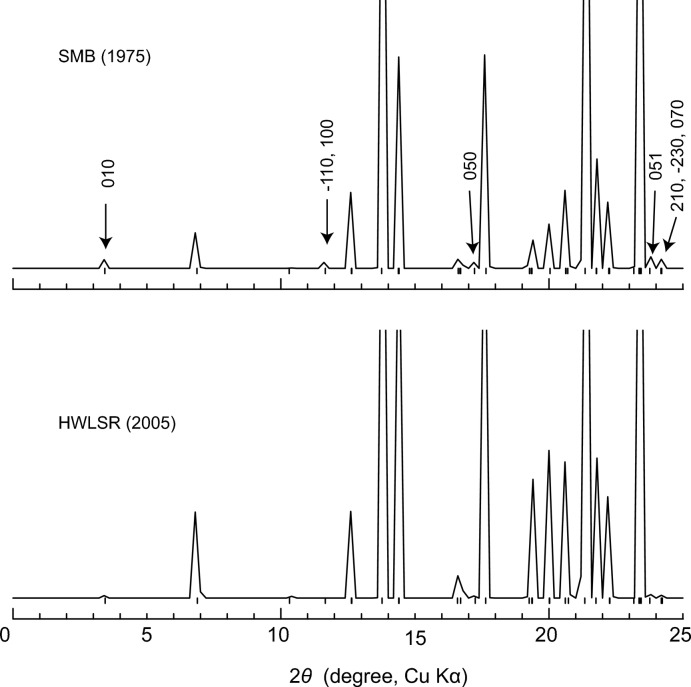
Powder diffraction diagrams of Nd2Ti2O7 calculated from the atomic coordinates given by Scheunemann & Müller-Buschbaum (1975) (top) and Harvey et al. (2005) (bottom). Indices are given for several peaks which characterise the difference between the two structures.

**Fig. 4.**
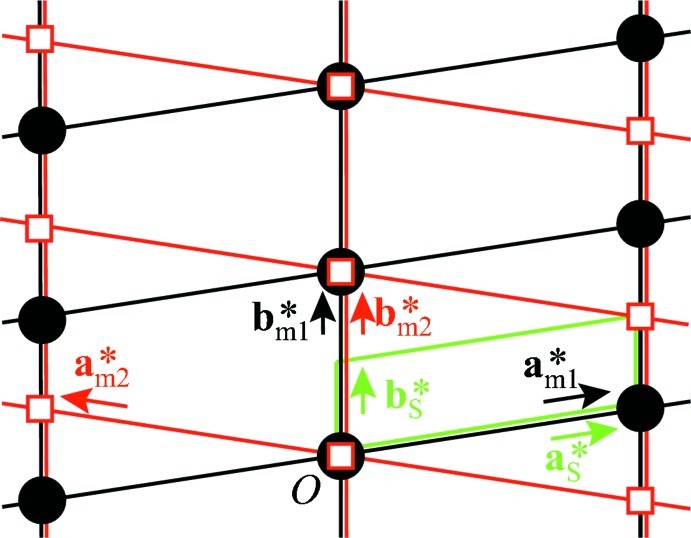
Schematic representation of the hk0 reciprocal section of Nd2Ti2O7. The monoclinic twin components, m1 and m2, are represented by black and red colour, respectively. The doubled monoclinic unit cell, S, determined by Scheunemann & Müller-Buschbaum (1975), and Harvey et al. (2005) is given in green colour. The c* axes of all modifications are perpendicular to the paper.

## supplementary crystallographic information

### Comment

The structure of Nd_2_Ti_2_O_7_ contains perovskite-type slabs consisting of TiO_6_ octahedra and Nd ions (Fig. 1 and Fig. 2(*a*)). The slabs are stacked along b with interconnecting Nd—O bonds. The thickness of the slabs corresponds to four corner-sharing TiO_6_ octahedra. The TiO_6_ octahedra have two tilt systems about c and b* which are closely related to the displacements of Nd atoms. The mode of the Nd atom displacements along b* is schematically illustrated by arrows in Fig. 2(*a*) with respect to the dashed lines drawn parallel to a. The Nd and Ti ions are displaced from the geometrical centres of respective coordination polyhedra so that a net polarization occurs in the crystal. The magnitude of the spontaneous polarization was estimated to be approximately 18 µC cm^-2^ along the polar *c* axis assuming formal charges for constituent atoms.

Scheunemann & Müller-Buschbaum (1975) (hereafter abbreviated as SMB) have previously determined the structure of Nd_2_Ti_2_O_7_ based on single-crystal X-ray diffraction data and reported monoclinic symmetry and space group *P*2_1_ with unit-cell dimensions *a* = 7.677 Å, *b* = 26.013 Å, *c* = 5.465 Å, and *γ* = 98.4° (the original cell setting in *P*12_1_1 has been converted to the current setting in *P*112_1_ with the *c* axis unique). The unit cell of the SMB structure (Fig. 2(*b*)) is doubled along b compared with that of the present study. The SMB structure contains two kinds of perovskite-type slabs with octahedra coloured in green and orange, respectively. The green-coloured slab is essentially the same as that in the present study, which can be understood in terms of the octahedral tilt about b* and the mode of the Nd atom displacements along b* as indicated by arrows. In contrast, the orange-coloured slab in the SMB structure has very small octahedral tilts about b* in combination with negligible Nd atom displacements along b*. The existence of different types of slabs stacked alternately along b justifies the doubled unit cell in the SMB structure. The presence of two modifications (Fig. 2(*a*) and (*b*)) at room temperature, having different unit cells with the same space group, leaves room for further investigations on the crystal chemistry of Nd_2_Ti_2_O_7_.

Harvey *et al.* (2005) (hereafter abbreviated as HWLSR) studied a Nd_2_(Zr_1-_*_x_*Ti*_x_*)_2_O_7_ solid solution and reported a monoclinic modification for the end member Nd_2_Ti_2_O_7_ based on neutron powder data and analyzed by the Rietveld method. The HWLSR structure is plotted in Fig. 2(*c*). The monoclinic unit cell of the HWLSR structure has similar dimensions to that of SMB. Although no reference is given in their paper to the initial structure model for the Rietveld refinement, it appears possible that the starting SMB model, if used, could easily converge to the HWLSR structure because the symmetry is the same and the parameter shifts are rather small. It is important, however, that the SMB and HWLSR structures are different in that the latter consists of essentially the same green-type slabs. The simulated powder diffraction diagrams of the SMB and HWLSR structures are shown in Fig. 3, indicating a differenece in intensities of reflections at low angles with *k* = odd. The structure-checking program *PLATON* (Spek, 2009) actually found additional twofold screw symmetries lying at the boundary of the slabs in the HWLSR structure with atom shifts less than 0.22 Å, hence suggesting a halved monoclinic unit cell as shown in Fig. 2(*a*).

Diffraction data in the present study were taken using a conventional sealed X-ray tube. Within the limitations of the experiment, we could not find any superstructure reflections that would double the unit cell volume. It should be noted that twinning of the investigated crystal does emerge as extra reflections with *h* = odd (Fig. 4), and that the reciprocal pattern of the components can be indexed on basis of doubled monoclinic unit cells like those of HWLSR or SMB. However, it is not difficult to distinguish the twinning because the extra extinction condition appears for reflections with *h* = even and *k* = odd (Fig. 4). A trial integration was carried out for the present crystal, assuming a doubled monoclinic cell similar like that of SMB or HWLSR, but the mode of Nd atom displacements in the doubled cell was essentially the same as those in Fig. 2(*c*), and no structure similar to Fig. 2(*b*) was obtained.

As a conclusion, the HWLSR structure model is supposedly the same as the present one. On the other hand, it seems difficult to discard the possibility of two monoclinic modifications for Nd_2_Ti_2_O_7_ at room temperature as far as the SMB structure exists.

### Experimental

Crystals were grown by the flux method using Nd_2_O_3_ (Wako Co., 99.9%) and TiO_2_ (Wako, 99%) as starting materials, and Na_2_MoO_4_.2H_2_O as the flux. A preliminary heat treatment at 1273 K for 12 h was applied to Nd_2_O_3_ before weighing. A 15 g mixture containing a 20 mol% solute corresponding to the Nd_2_Ti_2_O_7_ composition was put in a platinum crucible. The platinum crucible was then placed in an alumina crucible with alumina powder and heated in an electric furnace under air atmosphere. The sample was kept at 1373 K for 10 h, and then cooled to 1173 K at the rate of 5 K h^-1^, with subsequent furnace-cooling by turning off the power. Purple and transparent crystals with approximate dimensions of 200 × 300 × 50 µm^3^ were obtained after washing the flux component with warm water. Neither the flux component nor other impurities were detected beyond the limit of detection using energy dispersive spectroscopic analysis with a JED-2300 instrument (Jeol Ltd.).

### Refinement

The charge-flipping structure solution program *SUPERFLIP* (Palatinus & Chapuis, 2007) indicated that the present Nd_2_Ti_2_O_7_ crystals are isostructural with the monoclinic modifications of La_2_Ti_2_O_7_ (Gasperin, 1975) and Ca_2_Nb_2_O_7_ (Ishizawa *et al.*, 1980). Further refinements were thus carried out using the atom positions of monoclinic Ca_2_Nb_2_O_7_ as starting parameters. All the crystals showed twinning where one twin component (*m*2) is obtained by rotating the other (*m*1) by 180° about b*. The orientation relationships of the two twin components are illustrated in Fig. 4. The twin scheme is essentially the same as that described for La_2_Ti_2_O_7_ (Schmalle *et al.*, 1993). Since the monoclinic lattice is metrically the sublattice of the pseudo-orthorhombic one in reciprocal space (Fig. 4), all the reflections of the twin components with *h* = even are almost perfectly overlapped whereas those with *h* = odd are not overlapped at all. The integration of peak intensities were carried out separately for the lattices of the *m*1 and *m*2 components, and an absorption correction was processed by *TWINABS* (Bruker, 2008). In addition to the above, crystals undergo twinning by merohedry where the spontaneous polarization vectors of the ferroelectric domains are aligned oppositely along c. The refinement was thus carried out assuming four twin components, *m*1, *m*2 and their inverted ones, resulting in roughly similar volume fractions of 23 (3)%, 28 (2)%, 22 (1)% and 27 (1) %, respectively. The lowest angle reflections, 010 and its equivalents at *θ* =1.58°, were removed from the refinement because their intensities were seriously affected by the beamstop. Anisotropic and isotropic atomic displacement parameters were used for the two types of metal and the O atoms, respectively.

### Crystal data

**Table:**

Nd_2_Ti_2_O_7_	*F*(000) = 880
*M**_r_* = 496.2	*D*_x_ = 6.110 Mg m^−^^3^
Monoclinic, *P*112_1_	Mo *K*α radiation, λ = 0.71069 Å
Hall symbol: P 2zc	Cell parameters from 12720 reflections
*a* = 7.6747 (1) Å	θ = 3.2–40.2°
*b* = 13.0025 (2) Å	µ = 21.77 mm^−^^1^
*c* = 5.4640 (1) Å	*T* = 293 K
β = 90°	Block, purple
*V* = 539.24 (2) Å^3^	0.11 × 0.08 × 0.07 mm
*Z* = 4	

### Data collection

**Table:**

Bruker APEXII CCD diffractometer	6200 reflections with *I* > 3σ(*I*)
Radiation source: X-ray tube	*R*_int_ = 0.049
φ and ω scans	θ_max_ = 40.3°, θ_min_ = 1.6°
Absorption correction: multi-scan (*TWINABS*; Bruker, 2008)	*h* = −13→13
*T*_min_ = 0.134, *T*_max_ = 0.206	*k* = −23→23
25187 measured reflections	*l* = −9→9
6608 independent reflections	

### Refinement

**Table:**

Refinement on *F*	Weighting scheme based on measured s.u.'s *w* = 1/(σ^2^(*F*) + 0.0001*F*^2^)
*R*[*F*^2^ > 2σ(*F*^2^)] = 0.024	(Δ/σ)_max_ = 0.016
*wR*(*F*^2^) = 0.027	Δρ_max_ = 2.44 e Å^−^^3^
*S* = 1.37	Δρ_min_ = −1.57 e Å^−^^3^
6608 reflections	Extinction correction: B-C type 1 Gaussian isotropic (Becker & Coppens, 1974)
133 parameters	Extinction coefficient: 349 (12)
0 restraints	Absolute structure: Flack (1983), 3019 Friedel pairs
1 constraint	Flack parameter: 0.220 (12)

### Fractional atomic coordinates and isotropic or equivalent isotropic displacement parameters (Å^2^)

**Table:**

	*x*	*y*	*z*	*U*_iso_*/*U*_eq_	
Nd1	0.22817 (5)	0.906931 (15)	0.75507 (4)	0.00599 (5)	
Nd2	0.14522 (4)	0.574954 (15)	0.34489 (4)	0.00563 (5)	
Nd3	0.71953 (5)	0.881350 (15)	0.74678 (4)	0.00533 (5)	
Nd4	0.64866 (4)	0.612953 (17)	0.28408 (4)	0.00796 (5)	
Ti1	0.47020 (19)	0.87935 (5)	0.25800 (16)	0.00463 (15)	
Ti2	0.41480 (19)	0.67491 (5)	0.78904 (13)	0.00476 (16)	
Ti3	0.96662 (19)	0.88160 (5)	0.25934 (16)	0.00437 (15)	
Ti4	0.92426 (19)	0.67911 (5)	0.78238 (14)	0.00521 (16)	
O1	0.5306 (4)	0.9810 (2)	0.5246 (6)	0.0050 (6)*	
O2	0.5001 (5)	0.7695 (3)	0.4494 (7)	0.0081 (6)*	
O3	0.4073 (5)	0.5586 (2)	0.5773 (6)	0.0064 (5)*	
O4	0.2248 (6)	0.8923 (2)	0.3081 (6)	0.0099 (5)*	
O5	0.1746 (6)	0.6954 (2)	0.6923 (6)	0.0072 (5)*	
O6	0.4338 (5)	0.8188 (2)	0.9387 (6)	0.0044 (5)*	
O7	0.3756 (5)	0.6078 (3)	0.0680 (7)	0.0114 (7)*	
O8	0.9591 (5)	0.9796 (3)	0.5265 (6)	0.0079 (6)*	
O9	0.8867 (5)	0.7715 (3)	0.4553 (7)	0.0073 (6)*	
O10	0.8731 (5)	0.5702 (2)	0.5633 (6)	0.0065 (5)*	
O11	0.7275 (6)	0.9089 (2)	0.1730 (6)	0.0062 (5)*	
O12	0.6740 (6)	0.6933 (2)	0.8421 (6)	0.0084 (5)*	
O13	0.9747 (5)	0.8155 (2)	0.9432 (6)	0.0072 (6)*	
O14	0.9226 (5)	0.5940 (2)	0.0518 (6)	0.0063 (6)*	

### Atomic displacement parameters (Å^2^)

**Table:**

	*U*^11^	*U*^22^	*U*^33^	*U*^12^	*U*^13^	*U*^23^
Nd1	0.00488 (8)	0.00601 (7)	0.00701 (8)	0.00062 (9)	−0.00008 (13)	0.00075 (7)
Nd2	0.00502 (8)	0.00630 (7)	0.00558 (8)	0.00087 (10)	−0.00014 (11)	−0.00051 (6)
Nd3	0.00446 (8)	0.00581 (7)	0.00580 (8)	0.00100 (9)	−0.00004 (12)	0.00079 (6)
Nd4	0.00543 (9)	0.01077 (8)	0.00800 (9)	0.00231 (10)	−0.00042 (12)	−0.00119 (6)
Ti1	0.0050 (3)	0.0047 (2)	0.0043 (2)	0.0010 (4)	0.0009 (7)	0.0001 (2)
Ti2	0.0042 (3)	0.0046 (2)	0.0052 (3)	−0.0002 (3)	0.0013 (6)	−0.00020 (18)
Ti3	0.0041 (3)	0.0049 (2)	0.0044 (2)	0.0014 (3)	0.0011 (7)	0.0000 (2)
Ti4	0.0055 (3)	0.0045 (2)	0.0056 (3)	0.0003 (4)	0.0013 (7)	−0.0005 (2)

### Geometric parameters (Å, º)

**Table:**

Nd1—O1	2.691 (3)	Nd4—O9	2.710 (3)
Nd1—O1^i^	2.631 (3)	Nd4—O10	2.426 (4)
Nd1—O4	2.450 (3)	Nd4—O12^vi^	2.627 (3)
Nd1—O5	2.742 (3)	Nd4—O14	2.499 (4)
Nd1—O6	2.312 (4)	Ti1—O1	1.975 (3)
Nd1—O8^ii^	2.701 (4)	Ti1—O1^vii^	2.220 (3)
Nd1—O8^i^	2.659 (4)	Ti1—O2	1.812 (4)
Nd1—O11^i^	2.411 (3)	Ti1—O4	1.935 (5)
Nd1—O13^ii^	2.360 (4)	Ti1—O6^vi^	1.917 (3)
Nd2—O3	2.415 (4)	Ti1—O11	2.010 (4)
Nd2—O5	2.450 (3)	Ti2—O2	2.269 (4)
Nd2—O7	2.318 (4)	Ti2—O3	1.898 (3)
Nd2—O10^ii^	2.398 (4)	Ti2—O5	1.973 (4)
Nd2—O10^iii^	2.423 (3)	Ti2—O6	2.028 (3)
Nd2—O14^ii^	2.381 (4)	Ti2—O7^v^	1.760 (4)
Nd2—O14^iv^	2.457 (3)	Ti2—O12	1.989 (5)
Nd3—O1	2.410 (3)	Ti3—O4^viii^	1.984 (5)
Nd3—O2	2.621 (3)	Ti3—O8	1.944 (4)
Nd3—O4^i^	2.931 (3)	Ti3—O8^ix^	2.213 (3)
Nd3—O6	2.458 (4)	Ti3—O9	1.820 (4)
Nd3—O8	2.401 (3)	Ti3—O11	1.977 (4)
Nd3—O9	2.601 (4)	Ti3—O13^vi^	1.935 (3)
Nd3—O11^v^	2.355 (3)	Ti4—O5^viii^	1.964 (4)
Nd3—O12	2.474 (3)	Ti4—O9	2.196 (4)
Nd3—O13	2.494 (4)	Ti4—O10	1.851 (3)
Nd4—O2	2.636 (4)	Ti4—O12	1.984 (5)
Nd4—O3	2.472 (4)	Ti4—O13	1.965 (3)
Nd4—O3^iii^	2.481 (3)	Ti4—O14^v^	1.841 (3)
Nd4—O7	2.398 (4)		
			
O1—Ti1—O1^vii^	84.56 (13)	O4^viii^—Ti3—O8	88.84 (15)
O1—Ti1—O2	93.27 (15)	O4^viii^—Ti3—O8^ix^	83.46 (13)
O1—Ti1—O4	88.45 (14)	O4^viii^—Ti3—O9	101.10 (15)
O1—Ti1—O6^vi^	162.01 (13)	O4^viii^—Ti3—O11	164.50 (12)
O1—Ti1—O11	85.18 (13)	O4^viii^—Ti3—O13^vi^	93.06 (16)
O1^vii^—Ti1—O2	172.96 (16)	O8—Ti3—O8^ix^	85.77 (14)
O1^vii^—Ti1—O4	83.65 (13)	O8—Ti3—O9	91.99 (16)
O1^vii^—Ti1—O6^vi^	78.18 (12)	O8—Ti3—O11	86.75 (14)
O1^vii^—Ti1—O11	80.38 (13)	O8—Ti3—O13^vi^	165.40 (14)
O2—Ti1—O4	103.01 (15)	O8^ix^—Ti3—O9	174.89 (17)
O2—Ti1—O6^vi^	103.26 (15)	O8^ix^—Ti3—O11	81.40 (13)
O2—Ti1—O11	92.78 (15)	O8^ix^—Ti3—O13^vi^	80.08 (13)
O4—Ti1—O6^vi^	94.59 (16)	O9—Ti3—O11	93.89 (15)
O4—Ti1—O11	163.29 (13)	O9—Ti3—O13^vi^	101.82 (15)
O6^vi^—Ti1—O11	86.97 (15)	O11—Ti3—O13^vi^	87.61 (16)
O2—Ti2—O3	84.58 (13)	O5^viii^—Ti4—O9	86.72 (14)
O2—Ti2—O5	84.71 (13)	O5^viii^—Ti4—O10	90.78 (16)
O2—Ti2—O6	81.60 (13)	O5^viii^—Ti4—O12	167.53 (13)
O2—Ti2—O7^v^	172.61 (17)	O5^viii^—Ti4—O13	87.47 (15)
O2—Ti2—O12	81.44 (14)	O5^viii^—Ti4—O14^v^	100.64 (16)
O3—Ti2—O5	91.46 (16)	O9—Ti4—O10	82.06 (14)
O3—Ti2—O6	166.09 (13)	O9—Ti4—O12	82.86 (14)
O3—Ti2—O7^v^	98.63 (15)	O9—Ti4—O13	83.96 (14)
O3—Ti2—O12	95.54 (16)	O9—Ti4—O14^v^	171.88 (16)
O5—Ti2—O6	85.68 (14)	O10—Ti4—O12	94.53 (16)
O5—Ti2—O7^v^	101.79 (17)	O10—Ti4—O13	165.99 (15)
O5—Ti2—O12	163.81 (12)	O10—Ti4—O14^v^	94.32 (14)
O6—Ti2—O7^v^	95.28 (15)	O12—Ti4—O13	84.64 (15)
O6—Ti2—O12	84.04 (14)	O12—Ti4—O14^v^	90.22 (16)
O7^v^—Ti2—O12	91.59 (17)	O13—Ti4—O14^v^	99.66 (14)

Symmetry codes: (i) −*x*+1, −*y*+2, *z*+1/2; (ii) *x*−1, *y*, *z*; (iii) −*x*+1, −*y*+1, *z*−1/2; (iv) −*x*+1, −*y*+1, *z*+1/2; (v) *x*, *y*, *z*+1; (vi) *x*, *y*, *z*−1; (vii) −*x*+1, −*y*+2, *z*−1/2; (viii) *x*+1, *y*, *z*; (ix) −*x*+2, −*y*+2, *z*−1/2.
